# Pathophysiology of outer retinal corrugations: Imaging dataset and mechanical models

**DOI:** 10.1016/j.dib.2023.108920

**Published:** 2023-01-19

**Authors:** Isabela Martins Melo, Masoud Nourouzi Darabad, Arun Ramachandran, Paola Lourdes Oquendo, Hesham Hamli, Wei Wei Lee, Flavia Nagel, Aditya Bansal, Rajeev H. Muni

**Affiliations:** a Department of Chemical Engineering and Applied Chemistry, University of Toronto, Toronto, Ontario, Canada; b Department of Ophthalmology and Vision Sciences, University of Toronto, Toronto, Ontario, Canada; c Department of Ophthalmology, St. Michael's Hospital, Unity Health Toronto, Toronto, Ontario, Canada; d Faculty of Medicine, University of Toronto, Toronto, Ontario, Canada; e Kensington Vision and Research Institute, Toronto, Ontario, Canada

**Keywords:** Outer retinal corrugations, Outer retinal folds, Rhegmatogenous retinal detachment, Retinal pigment epithelium control, Outer retinal modulus of elasticity

## Abstract

This article presents high-resolution swept-source optical coherence tomography (SS-OCT) imaging data used to elaborate a mechanical model that elucidates the formation of outer retinal corrugations (ORCs) in rhegmatogenous retinal detachments (RRD). The imaging data shared in the repository and presented in this article is related to the research paper entitled “Outer Retinal Corrugations in Rhegmatogenous Retinal Detachment: The Retinal Pigment Epithelium-Photoreceptor Dysregulation Theory” (Muni et al., AJO, 2022). The dataset consists of 69 baseline cross-sectional SS-OCT scans from 66 patients that were assessed for the presence of ORCs and analyzed considering the clinical features of each case. From the 66 cases, we selected SS-OCT images of 4 RRD patients with visible ORCs and no cystoid macular edema (CME) to validate the mechanical model. We modelled the retina as a composite material consisting of the outer retinal layer (photoreceptor layer) and the inner retinal layer (the part of the retina that excludes the photoreceptor layer) with thicknesses $T_{o}$ and $T_{i}$ and elastic modulus $E_{o}$ and $E_{i}$, respectively. The thickness of the outer and inner retinal layers and the relative increase in the length of the outer retinal layer (γ) were measured from the SS-OCT images. Measurements from the SS-OCT images of patients with RRD demonstrated a 30% increase (γ=0.3) in the length of the outer retinal layer and a 400% increase in the thickness of the outer retinal layer (To). Using the mathematical model, Eo/Ei ranged between 0.05 to 0.5 to result in ORCs with a similar frequency to those observed in the SS-OCT scans.

**Specifications Table**

**Table:** 

Subject	Ophthalmology.
Specific subject area	Outer retinal corrugations; Rhegmatogenous retinal detachment.
Type of data	Image
How the data were acquired	Patients with rhegmatogenous retinal detachment underwent imaging with the Zeiss PLEX Elite 9000 SS-OCT using high-definition horizontal spotlight 16mm scan (× 100), high-definition horizontal 51-line 12mm scan and 12 × 12-mm macular cube, and, in addition, Optos Silverstone ultra-widefield guided SS-OCT (UWF-SS-OCT), depending on the location of the detachment. A subset of images was used to extract input data for mechanical models that determined the elastic energy required to generate outer retinal corrugations with the observed frequency.
Data format	Raw
Description of data collection	Patients with primary rhegmatogenous retinal detachment who presented to the tertiary vitreoretinal surgery practice at St. Michael's Hospital, Unity Health Toronto, Toronto, Ontario, Canada from August 2020 to February 2022 underwent baseline imaging with the PLEX Elite 9000 SS-OCT.
Data source location	Department of Ophthalmology, St. Michael's Hospital/Unity Health Toronto 8th floor, Donnelly Wing St. Michael's Hospital 30 Bond St., Toronto, Ontario, M5B 1W8
Data accessibility	Raw SS-OCT images and input variables and output results from the mechanical models have been uploaded to a data repository: Martins Melo, Isabela; Darabad, Masoud; Ramachandran, Arun et al. (2022), “Outer Retinal Corrugations: Imaging Dataset and Mechanical Models”, Mendeley Data, v2 doi:10.17632/bzsc7gd9p3.2 *https://data.mendeley.com/datasets/bzsc7gd9p3/2*
Related research article	Muni RH, Darabad MN, Oquendo PL, Hamli H, Lee WW, Nagel F, Bansal A, Melo IM, Ramachandran A. Outer Retinal Corrugations in Rhegmatogenous Retinal Detachment: The Retinal Pigment Epithelium-Photoreceptor Dysregulation Theory. Am J Ophthalmol. 2022 Sep 5;245:14-24. doi:10.1016/j.ajo.2022.08.019. Epub ahead of print. PMID: 36067852.

**Value of the Data**

• Following rhegmatogenous retinal detachment there are several structural changes that occur to the retina, which can subsequently result in post-operative anatomic abnormalities and worse functional outcomes. This dataset helps us understand what happens to the mechanical properties of the retina that result in the occurrence of outer retinal corrugations.
• Professionals in Ophthalmology/Retina and engineers could use this imaging dataset and corresponding data/mechanical models to study other conformational changes that occur in the structure of the retina in the setting of rhegmatogenous retinal detachment.
• Outer retinal corrugations (ORCs) that do not resolve result in outer retinal folds and worse functional outcomes. Thus, understanding the pathogenesis of ORCs may lead to modifications in retinal detachment repair concepts and techniques that favor the resolution of ORCs prior to contact of the retina with the retinal pigment epithelium.

## 1 Objective

This imaging dataset is related to an original research article (Muni et al., AJO 2022) [1] and allowed us to explore the pathophysiology of outer retinal corrugations (ORCs), a very common clinical finding in RRD. ORCs can lead to the formation of ORFs and worse postoperative visual outcomes. The detailed analysis of these images informed the development of a mechanical model that characterizes the pathophysiological process by which ORCs develop. Measurements performed on the images resulted in input values that were used to calculate the elasticity of the detached inner and outer retina (E_i_ and E_o_, respectively) and the necessary energy that would be required to bend the tissue with the observed frequencies.

## 2 Data Description

These imaging data consist of real-time Swept-Source Optical Coherence Tomography (SS-OCT) scans that demonstrate morphological characteristics of retinal detachments with and without outer retinal corrugations (ORCs). The ORCs observed in these SS-OCT images are a visual representation of RPE-photoreceptor dysregulation in patients with extensive and progressive detachments, a concept described in the original research article (Muni et al., AJO 2022) [1].

## 3 Experimental Design, Materials and Methods

This was a prospective cohort study of patients with primary rhegmatogenous retinal detachment (RRD) who presented to the tertiary vitreoretinal surgery practice at St. Michael's Hospital, Unity Health, Toronto, Ontario, Canada from August 2020 to February 2022. To assess the occurrence of outer retinal corrugations (ORCs), baseline imaging was performed in every patient as described by Muni et al. [1] using Carl Zeiss PLEX® Elite 9000 Swept Source Optical Coherence Tomograph (SS-OCT) and Optos Silverstone ultra-widefield guided SS-OCT. Scans were acquired with the tracker off in fovea-involving detachments, with either the 51-line protocol with 50 times averaging, which produces a scan of 12 mm in length, or the 1-line HD-spotlight scan with 100 times averaging, which produces a scan of 16 mm in length. When using the UWF-SS-OCT, scans were acquired using the 23 mm extended line or a 6 mm single-line of the HD-volume. The primary outcome of the study was to understand the pathophysiology underlying the formation of ORCs in humans *in vivo* with the assistance of SS-OCT.

**Fig. 1:**
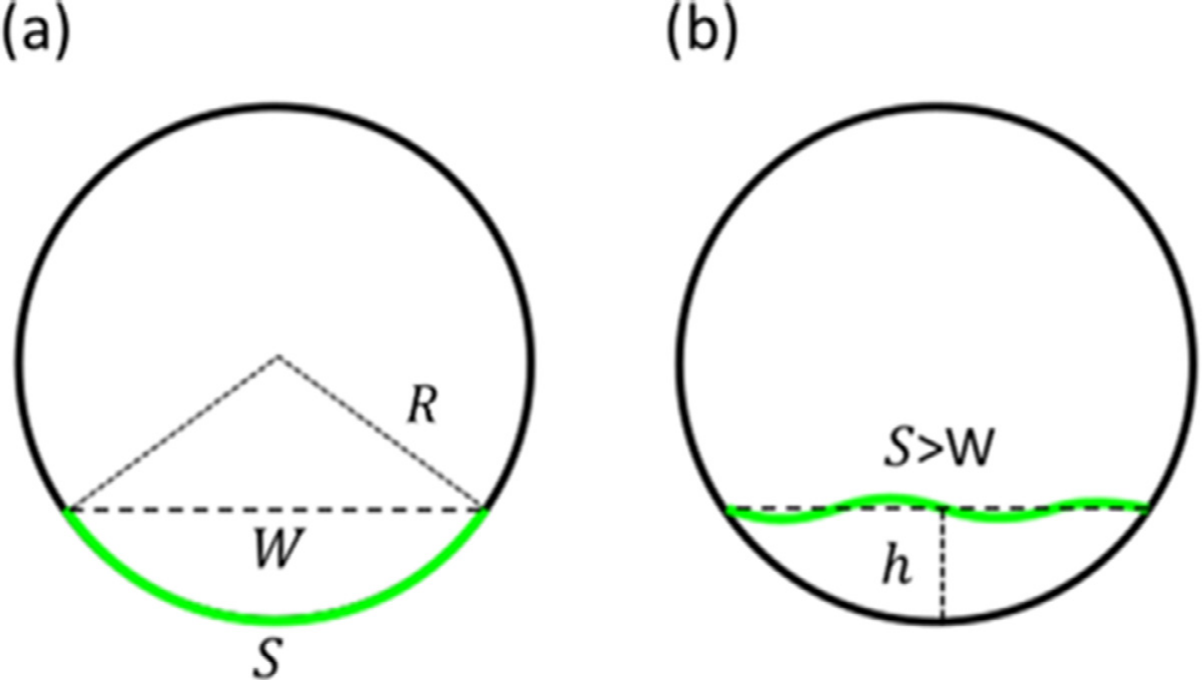
Schematic representation of the detachment height in relation to the eye sphere. (a) A two-dimensional schematic that represents a part of the retina with length S, and chord length W attached to the RPE. (B) A detachment with height $h=h_{c}/2,$ the retinal tissue of length S, needs to be accommodated in a space with length W, which results in the buckling of the retina.

66 cross-sectional OCT scans were assessed for the presence of ORCs and analyzed considering the clinical features of each case, to determine the presence of RPE-photoreceptor dysregulation. From the 66 cases, a sub-set of SS-OCT images was used to deduce the mechanical properties that are likely to lead to conformational changes in the retina upon detachment. ORC mathematical models are explained in detail below:

Ultra-widefield fundus imaging shows that the retina typically exhibits full-thickness undulations, where the outer and inner retina deform symmetrically. To understand this, recall that the healthy retina is affixed to the retinal pigment epithelium (RPE), which is a nearly spherical surface of radius, say R. Consider an arc of the retina with an arc length S and chord length W attached to the RPE, as depicted in the 2-dimensional schematic in Fig. 1(a). For any detachment with height hsmaller than a critical height $h<h_{c}$ where $h_{c}=2\left(R-\sqrt{R^{2}-W^{2}/4}\right)$, the detached part of the retina needs to be accommodated in a smaller projected area and thus always experiences a redundancy. When a thin sheet is subjected to restrictions that attempt to decrease its area, its response is to undergo bending instabilities such as buckling or wrinkling beyond a critical compressive stress [2] (see Section 1 for calculation details of the critical compressive stress needed to buckle the retina). Thus, any detachment with height $h<h_{c}\;$is expected to lead to full-thickness undulations in the retina. For example, for a detachment with height $h=\frac{h_{c}}{2}$ in Fig. 1(b), the retinal tissue of length S, needs to be accommodated in a space with length W<S, which results in undulations. For an extreme case, where the detachment spans half of the axial length eye, the critical height is equal to the diameter of the eye ($h_{c}=2R$); such detachment heights are usually less common in clinical practice.

**Fig. 2:**
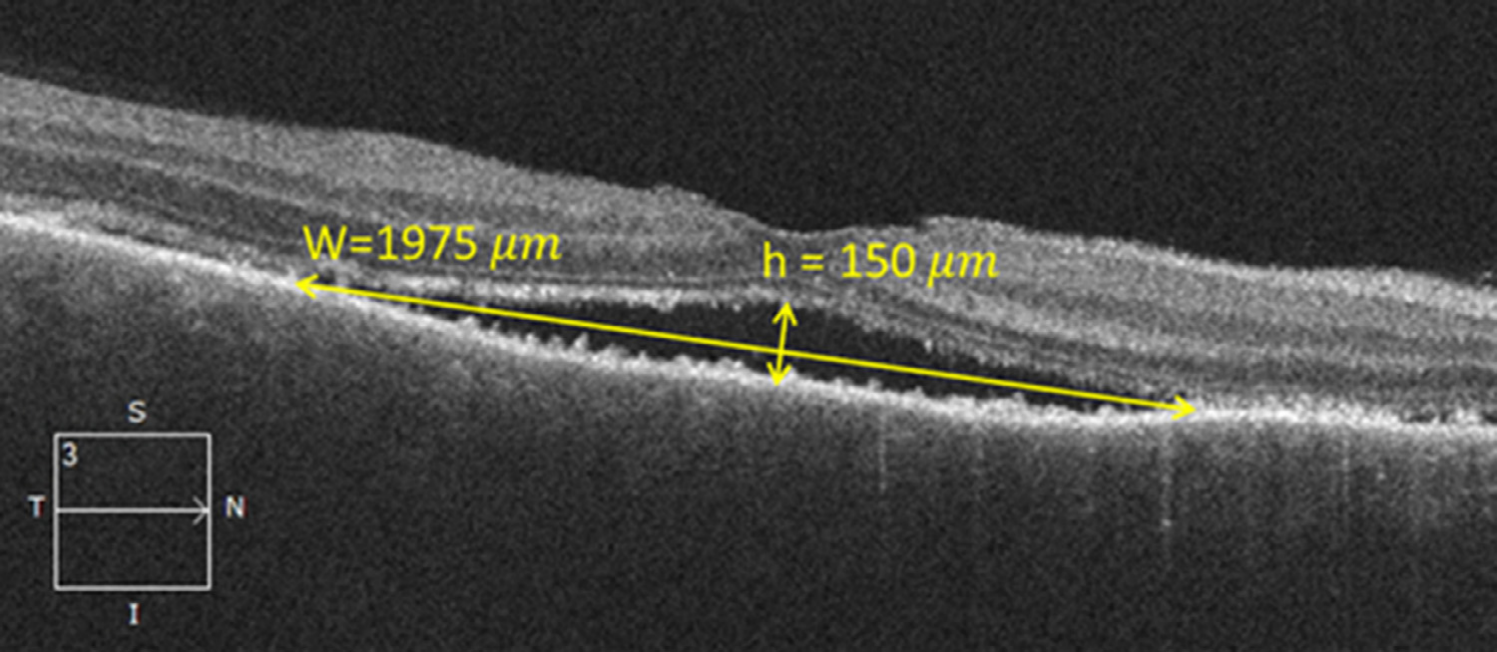
Detachment height and basal diameter measured in optical coherence tomography scan. Swept-source optical coherence tomography (SS-OCT) scan showing an example of an exudative retinal detachment with chord length W=1975μm, and height h=150. Assuming that the radius of the eye is R∼11200μm, we calculate a critical height $h_{c}∼78\;\mu m$. This suggests that the retinal tissue is under stretch.

For detachments with heights greater than the critical height ($h>h_{c})$, the detached portion of the retina will experience a stretch in all directions, and a membrane under tension does not display undulations. This mostly happens for detachments with small basal dimensions, as the corresponding critical heights are also small. An example of stretched retina is presented in the OCT image in Fig. 2, showing a localized small exudative retinal detachment with chord length W=1975μm and detachment height h=150μm. Assuming that the radius of the eye is R∼11200μm, we calculate the critical height to be $h_{c}=78\;\mu m$, which is less than the detachment height; we conclude, therefore, that the retina is under stretch in this example.

**Fig. 3:**
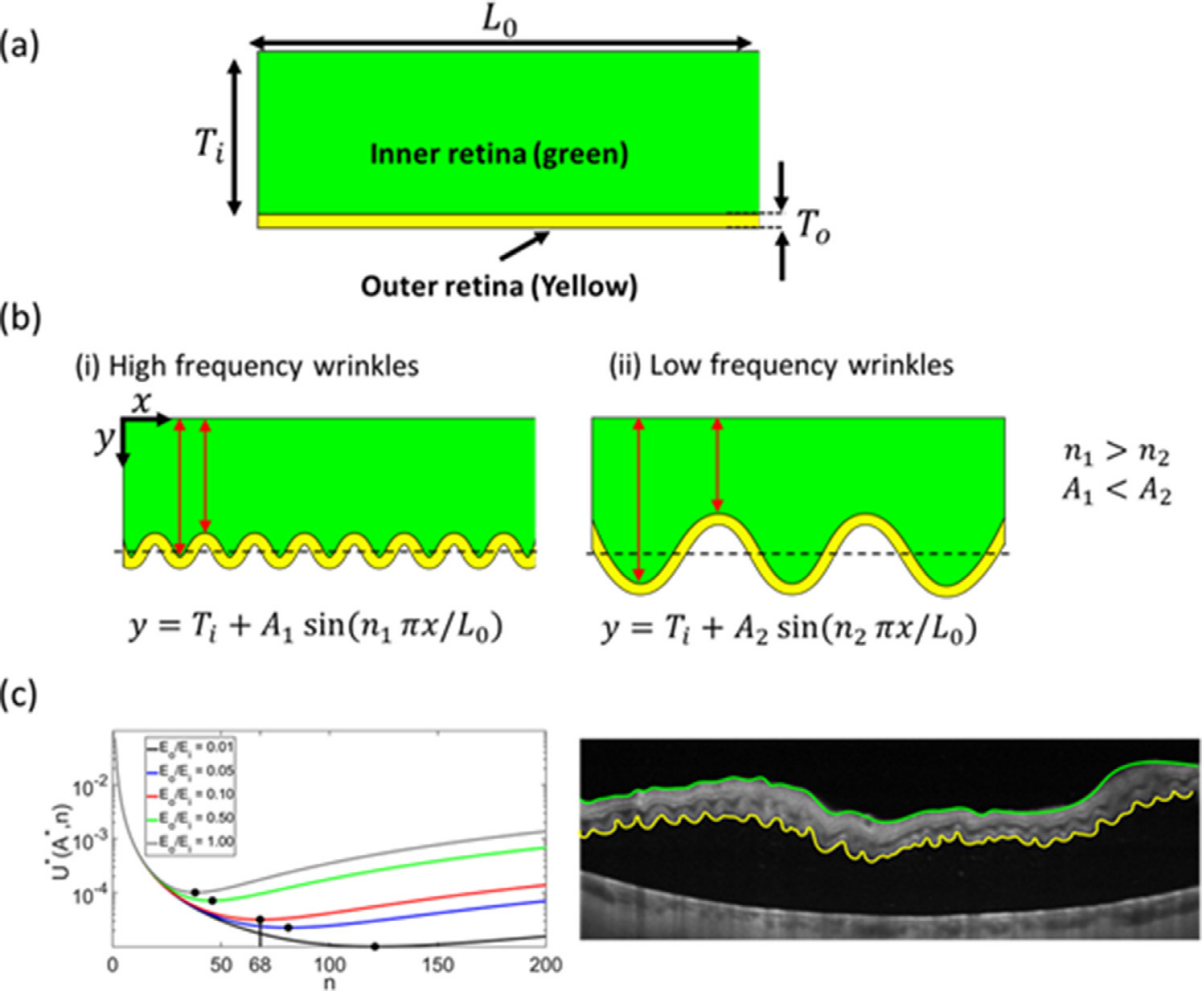
Mechanical model applied to outer retinal corrugations observed in optical coherence tomography scan. (a) The undeformed retina of lateral length $L_{0}$ comprising an inner retina of thickness $T_{i}$ (green), and an outer retina of thickness $T_{o}$. (b) Deformed retina with high frequency (i) or low-frequency corrugations (ii) in the outer retina, which also induces elastic stretching and compression in the inner retina. (c) The plot of the energy versus number of corrugations (n) for the optical coherence tomography (OCT) image calculated for various values of $E_{o}/E_{i}$. $L_{i}=2250\;\mu m$ is estimated from the length of inner retina measured from OCT along the green line. The length of the outer retina under corrugations $L_{o}=2940\;\mu m$ is measured along the yellow line from the OCT. The number of the corrugations in the OCT image is $n_{obs}∼65$. Our calculations show that the $\frac{E_{o}}{E_{i}}∼0.1$ to observe a similar number of corrugations in the outer retina (red line in the plot).

**Fig. 4:**
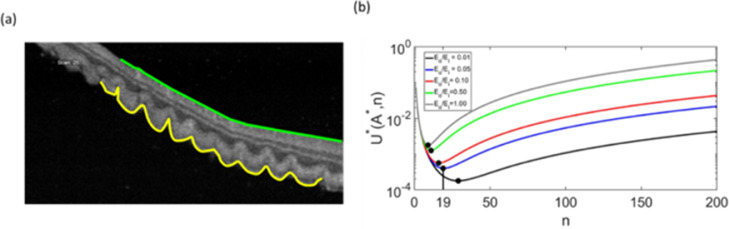
Mechanical model applied to outer retinal corrugations observed in optical coherence tomography scan. (a) Optical coherence tomography (OCT) image of patient with RRD, with $L_{i}=974\;\mu m$ (measured along the green line) and $L_{o}=1270\;\mu m$(measured along the yellow line), $T_{o}=65\pm 2\;\mu m$, $T_{i}=86\pm 8\;\mu m$, and $n_{obs}\approx 20$ (b) Plot Shows the total energy of the corrugated retina for various ratios of $E_{o}/E_{i}$. Our analysis in Fig. 4b suggests that the $E_{0}/E_{i}∼0.05$$E_{o}/E_{i}∼0.5$ can produce corrugations with a frequency similar to what has been observed on the OCT image.

**Fig. 5:**
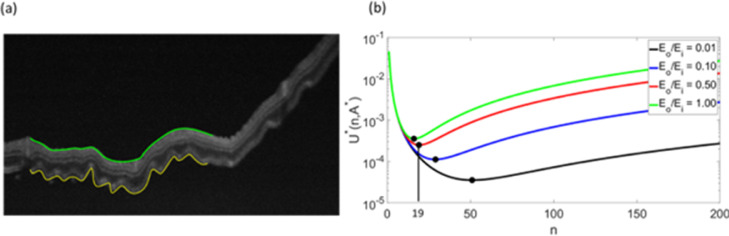
Mechanical model applied to outer retinal corrugations observed in optical coherence tomography scan. (a) Optical coherence tomography (OCT) image of a patient with RRD, with $L_{i}=3745\;\mu m$ and $L_{o}=4527\;\mu m$ (measured along the green and yellow lines, respectively) and the thicknesses of $T_{i}=351\pm 60\;\mu m$ and *To=*106±11μm, and $n_{obs}\approx 19$. (b) Plot Shows the total energy of the corrugated retina for various ratios of $E_{o}/E_{i}$. Our analysis in Fig. 5b suggests that the $E_{0}/E_{i}∼0.5$$E_{o}/E_{i}∼0.5$ can produce corrugations with a frequency similar to what has been observed on OCT image.

**Fig. 6:**
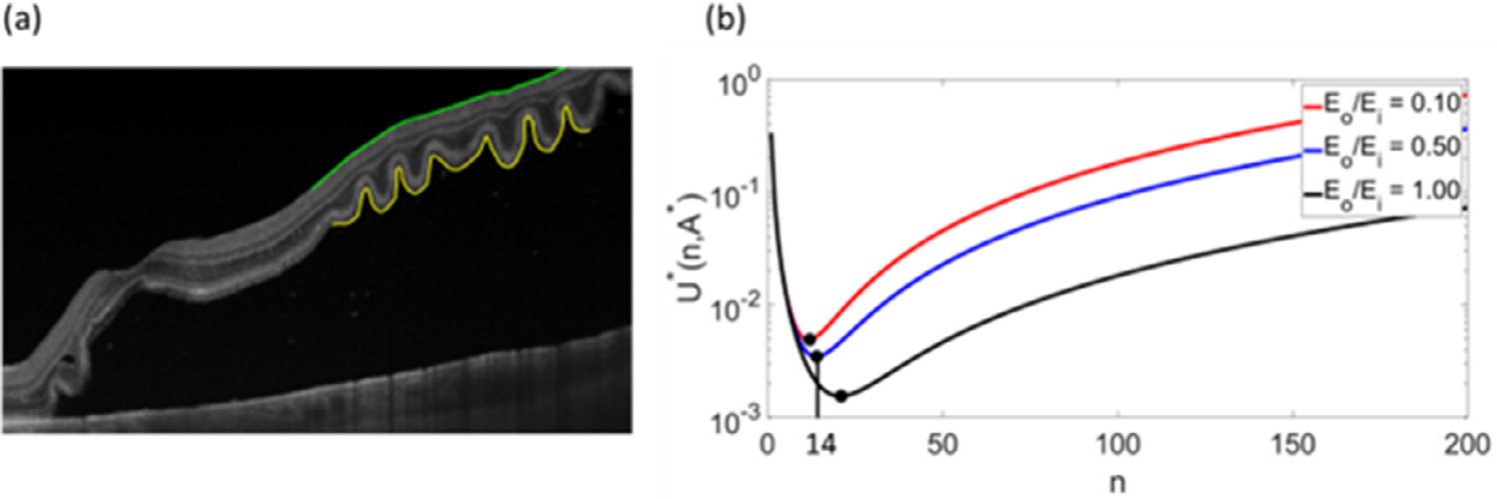
Mechanical model applied to outer retinal corrugations observed in optical coherence tomography scan. (a) Optical coherence tomography (OCT) image of a patient with RRD, with $L_{i}=3676\;\mu m$ and $L_{o}=7288\;\mu m$ (measured along the green and yellow lines, respectively) and the thicknesses of $T_{i}=501\pm 190\;\mu m$ and *To* = 19±20μm, and $n_{obs}\approx 14$. (b) Plot Shows the total energy of the corrugated retina for various ratios of $E_{o}/E_{i}$. Our analysis in Fig. 4b suggests that the $E_{0}/E_{i}∼0.5$ can produce corrugations with a frequency similar to what has been observed on the OCT image.

In the ORC phenomenon, high-frequency corrugations (shorter wavelength) in the outer retina are superimposed on the long wavelength undulations of the full-thickness retina. This suggests that the pathophysiology of ORCs could involve processes that result from differences in the mechanical properties of the outer and inner retina in RRD. To estimate the difference in Young's elastic moduli of the outer and inner retina in the detached state ($E_{o}$ and $E_{i}$, respectively), we devised a mathematical model based on prior work [3], [4], [5], [6] (calculation details can be found in section 2). Briefly, we consider the retina to be a composite material comprised of layers of the outer retina (photoreceptor layer) and the inner retina (the retinal thickness that excludes the photoreceptor layer), with thicknesses of $T_{o}$ and $T_{i}$, respectively Fig. 3(a). Since the outer retina shows high-frequency corrugations on the OCT images, we model the mechanical response of this region using a bending energy contribution. The corrugations or wrinkles compress and stretch the inner retina along its thickness, which leads to an elastic energy contribution from the inner retinal layer (Fig. 3(b)). Both energies are unfavourable (positive), as the base state for both layers is a flat shape. For a given lateral expansion of the outer retina, the final frequency of the ORCs is determined by a competition between the bending energy of the outer retina and the elastic energy of the inner retina. Very high frequency (short wave) ORCs would result in small amplitudes and low elastic stretching energy but are accompanied by a high bending energy contribution of the outer retina due to the sharp curves (Fig. 3(b) (i)). On the other hand, very low-frequency ORCs would lead to large amplitudes and are prohibited by a high elastic energy contribution from the inner retina [see Fig. 3(b) (ii)]. Thus, there is an optimum frequency where the energy is minimized, and the system mechanically deforms to this frequency. Our analysis of the OCT images (see Fig. 3(c) and Fig. 4, Fig. 5, Fig. 6 for more examples), indicates that $E_{o}/E_{i}$ range from 0.05 to 0.5 for the manifestation of the observed ORC frequencies.

### 3.1 Section 1 – critical stress for buckling of the retina

Here, we use the Euler's equation for critical buckling load applied to thin films, to estimate the critical stress $\sigma_{cr}$ needed to buckle the retina. Assuming a rectangular cross section of the retina with length a and width b and thickness t:

$$\sigma_{cr}=k_{cr}\frac{\pi^{2}E}{12\left(1-\nu^{2}\right){\left(\frac{b}{t}\right)}^{2}}$$

In this expression, $k_{cr}$ is buckling coefficient that depends on the geometry of the thin film:

$$k_{cr}=\left(\frac{mb}{a}+\frac{a}{mb}\right)$$

where m is the number of half sine waves that occurs lengthwise. Assuming a∼b∼50t and m=1, we find $\sigma_{cr}=30\;Pa$.

### 3.2 Section 2. A mathematical model for outer retinal corrugations

Consider a membrane (outer retina) of bending modulus $k_{b}$ of length $L_{0}$ attached to a substrate (inner retina) of length $L_{0}$, thickness $T_{i}$ and young's modulus $E_{i}$. We assume that the outer retina is thin compared to the inner retina (a gross approximation). If the membrane shape is perturbed to the shape $y=T_{i}+h\left(x\right)$ due to an increase in length of the outer retina to L,then two changes in the energy of the composite can be expected. First, the inner retinal substrate is stretched and compressed at different locations, which leads to an elastic energy contribution. Second, the outer retina is subjected to bending stresses as it changes its shape; this leads to a bending contribution. The total energy is, thus,

$$U=\int_{0}^{L_{0}}\frac{E_{i}h^{2}}{T_{i}}dx+\int_{0}^{L_{0}}k_{b}{\left\{\frac{\frac{d^{2}h}{dx^{2}}}{{\left[1+{\left(\frac{dh}{dx}\right)}^{2}\right]}^{\frac{3}{2}}\;}\right\}}^{2}dx,$$

where the first term represents the contribution from elastic energy of the inner retina and second term represents the contribution from bending stiffness of the outer retina. Also, there is a geometric constraint that needs to be satisfied - the length Lof the outer retina, which is greater than $L_{0}.$

$$L=\int_{0}^{L_{0}}\sqrt{1+{\left(\frac{dh}{dx}\right)}^{2}}dx.$$

Assuming a sinusoidal form for the perturbation in the shape, $h=A\sin(\frac{n\pi x}{L_{0}})$, where Ais the amplitude and n is the number of half waves of the perturbed outer retina over the length $L_{0}$, the expression for total energy is transformed to

$$U=\frac{E_{i}A^{2}}{T_{i}}\int_{0}^{L_{0}}\sin^{2}\left(\frac{n\pi x}{L_{0}}\right)dx+\frac{k_{b}A^{2}n^{4}\pi^{4}}{L_{0}^{4}}\int_{0}^{L_{0}}\frac{\sin^{2}\left(\frac{n\pi x}{L_{0}}\right)}{{\left[1+\frac{A^{2}n^{2}\pi^{2}}{L_{0}^{2}}\cos^{2}\left(\frac{n\pi x}{L_{0}}\right)\right]}^{3}\;}dx,$$

and the geometrical constraint becomes

$$L=\int_{0}^{L_{0}}\sqrt{1+\frac{A^{2}n^{2}\pi^{2}}{L_{0}^{2}}\cos^{2}\left(\frac{n\pi x}{L_{0}}\right)}dx.$$

Rendering the equations dimensionless, we derive

$$U^{*}\left(A^{*},n\right)=A^{*2}\int_{0}^{1}\sin^{2}\left(n\pi x^{*}\right)dx^{*}+\beta A^{*2}\int_{0}^{1}\frac{\sin^{2}\left(n\pi x^{*}\right)}{{\left[1+n^{2}\pi^{2}A^{*2}\cos^{2}\left(n\pi x^{*}\right)\right]}^{3}\;}dx^{*},$$

with the dimensionless constraint,

$$L^{*}=1+\gamma =\int_{0}^{1}\sqrt{1+n^{2}\pi^{2}A^{*2}\cos^{2}\left(n\pi x^{*}\right)}dx^{*}.$$

Here,

$$x^{*}=\frac{x}{L_{0}},\;A^{*}=\frac{A}{L_{0}},\;U^{*}=\frac{U}{\frac{E_{i}L_{0}^{3}}{T_{i}}},$$

and

$$\beta =\frac{n^{4}\pi^{4}k_{b}T_{i}}{E_{i}L_{0}^{4}}.$$

The parameter γ is the relative change in length of the outer retinal layer, i.e. $\gamma =\left(L-L_{0}\right)/L_{0}$. Also, the bending stiffness of the outer retina $k_{b}$ is related to $T_{0}\;$thickness of the outer retina and its elastic modulus $E_{o}$ as $k_{b}=\frac{E_{o}T_{o}^{3}}{12(1-\nu^{2})}$.

To find the optimum value of n and corresponding optimum amplitude A of corrugations for a given ratio $E_{o}/E_{i},$ we directly evaluate the energy term for a range of values of n∈{1,2,…,200}. For a given n, we calculate the corresponding amplitude A(n) by solving the geometric constraint equation. Then the (n,A(n)) pair is used to evaluate the total energy expression. The total energy is then plotted over the given range of value of n to find the optimal values of $n_{opt}$ and $A_{opt}$.

## Ethics Statements

Ethics Committee approval was obtained from St. Michael's Hospital/Unity Health Toronto (REB 18-018), all participants underwent informed consent by the research coordinator, and the study was carried out in accordance with the Declaration of Helsinki.

## CRediT authorship contribution statement

**Isabela Martins Melo:** Methodology, Writing – original draft, Writing – review & editing. **Masoud Nourouzi Darabad:** Methodology, Software, Validation, Writing – original draft. **Arun Ramachandran:** Conceptualization, Methodology, Software, Validation. **Paola Lourdes Oquendo:** Data curation, Methodology. **Hesham Hamli:** Data curation, Methodology. **Wei Wei Lee:** Data curation, Methodology. **Flavia Nagel:** Data curation, Methodology. **Aditya Bansal:** Data curation, Methodology. **Rajeev H. Muni:** Investigation, Conceptualization, Writing – original draft, Writing – review & editing, Supervision.

## Declaration of Competing Interest

The authors declare that they have no known competing financial interests or personal relationships that could have appeared to influence the work reported in this paper.

## Data Availability

Outer Retinal Corrugations: Imaging Dataset and Mechanical Models (Original data) (Mendeley Data).
